# RNA binding protein ILF3 increases CEP55 mRNA stability to enhance malignant potential of breast cancer cells and suppress ferroptosis

**DOI:** 10.1186/s41065-025-00372-0

**Published:** 2025-01-27

**Authors:** Sheng Chen, Yangyong Luo, Simin Ruan, Guosen Su, Guoxing Huang

**Affiliations:** Department of Breast Disease, GaoZhou People’Hospital, Guangdong Province 89 Xiguan Road, Maoming City, Gaozhou City, 525200 China

**Keywords:** Breast cancer, Ferroptosis, CEP55, RNA binding protein, ILF3

## Abstract

**Background:**

Ferroptosis has emerged as a promising therapeutic target in cancer treatment. CEP55, a key regulator of cell mitosis, plays a significant role in the tumorigenesis of many malignancies. In this study, we elucidated the function of CEP55 in the ferroptosis of breast cancer (BC).

**Methods:**

The protein levels of CEP55 and ILF3 were detected by immunoblotting or immunohistochemistry, and their mRNA levels were assessed by quantitative PCR. Cell invasion and migration were evaluated by transwell assay. Cell apoptosis and colony formation were tested by flow cytometry and colony formation assays, respectively. RNA immunoprecipitation (RIP) experiment and CEP55 mRNA stability assay were used to validate the relationship between ILF3 and CEP55 mRNA. Subcutaneous xenograft studies were performed to analyze the role of ILF3 depletion in tumor growth.

**Results:**

CEP55 and ILF3 were upregulated in most of human BC samples and MDA-MB-231 and MCF-7 BC cells. The depletion of CEP55 or ILF3 impaired the growth, invasion, and migration of MDA-MB-231 and MCF-7 cells, while promoted their ferroptosis and apoptosis. Mechanistically, ILF3 stabilized CEP55 mRNA to regulate CEP55 expression in BC cells. CEP55 restoration partially rescued the malignant potential defects of ILF3-depleted BC cells and attenuates their ferroptosis. Moreover, ILF3 depletion enhanced the anti-tumor growth activity of the ferroptosis inducer erastin in MDA-MB-231 subcutaneous xenograft tumors.

**Conclusion:**

Our observations indicate that the depletion of ILF3 impairs the malignant potential of BC cells and promotes their ferroptosis by downregulating CEP55 expression. Silencing ILF3 or CEP55 could represent a potential therapeutic strategy for BC treatment.

**Graphical abstract:**

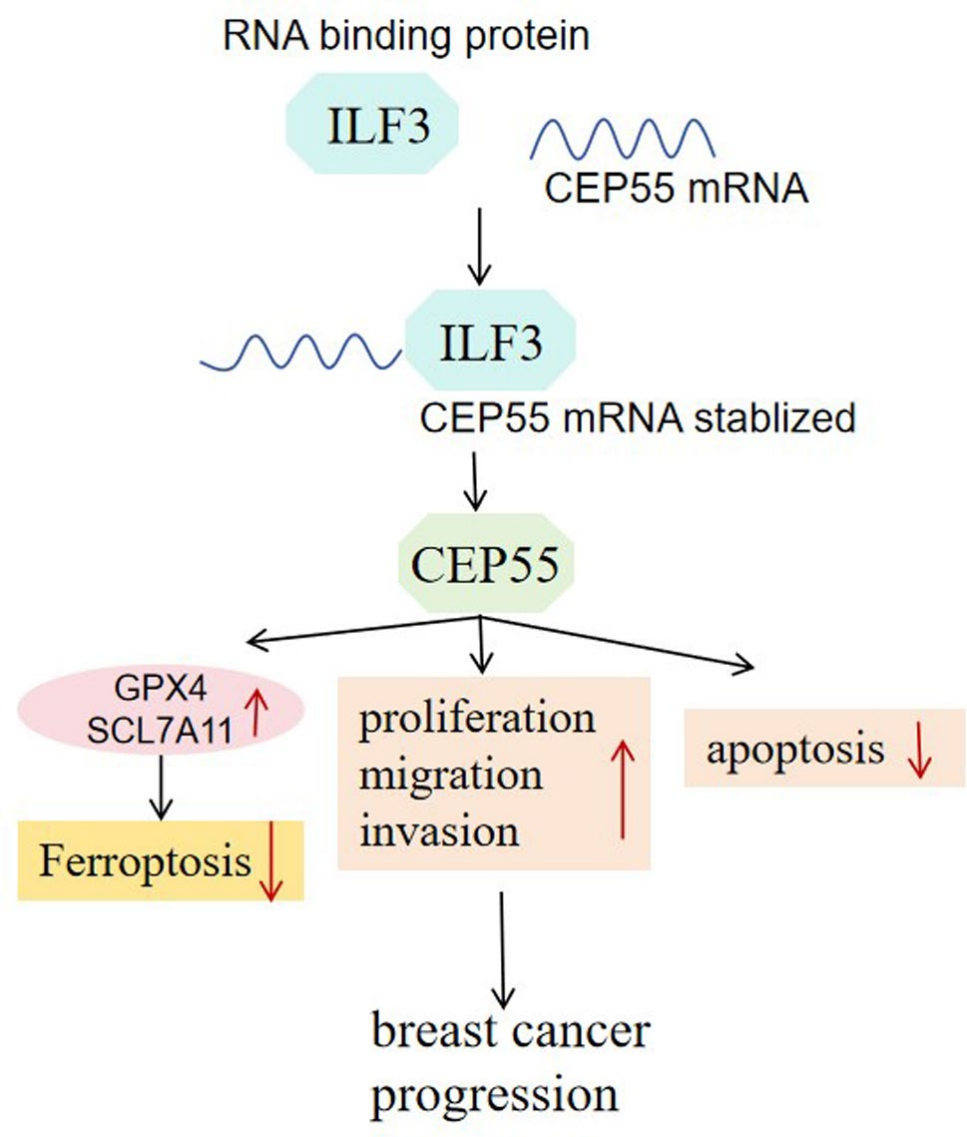

**Supplementary Information:**

The online version contains supplementary material available at 10.1186/s41065-025-00372-0.

## Highlights

ILF3 stabilizes CEP55 mRNA to regulate CEP55 expression.

ILF3 depletion enhances the anti-growth activity of erastin in subcutaneous xenografts.

The ILF3/CEP55 axis suppresses ferroptosis to promote BC progression.

**Supplementary Information:**

The online version contains supplementary material available at 10.1186/s41065-025-00372-0.

## Introduction

Breast cancer (BC), which originates from the epithelial or ductal cells of the breast, poses a significant global health challenge. Among women, it is the most prevalent cancer diagnosis, with an estimated 2.3 million new cases and 666,000 deaths globally in 2022, making it the leading female malignancy in terms of both incidence and mortality rates [1]. This disease is multifaceted, with its etiology intricately linked to various factors, including hormonal impacts, genetic predispositions, menstrual and reproductive patterns, atypical breast hyperplasia, and exposure to ionizing radiation [2]. Currently, the management of BC involves a diverse array of treatments, including surgical intervention, chemotherapy, endocrine modulation, and radiation therapy. However, despite significant advancements, conventional treatment methods have reached a plateau in their effectiveness and are often associated with considerable toxic adverse effects [3–5]. To advance personalized targeted therapies, a better understanding of the mechanisms governing BC initiation and progression is essential.

Ferroptosis, a newly recognized form of iron-dependent programmed cell death, has garnered substantial attention in cancer research in recent years [6, 7]. Research into ferroptosis in BC has made significant strides, elucidating its crucial roles across various subtypes and highlighting its potential as a therapeutic strategy [8]. Studies have shown that inducing ferroptosis can be an effective method for treating refractory BC, particularly triple-negative BC [9, 10]. A deeper understanding of the diverse mechanisms of ferroptosis in BC has paved the way for the discovery of innovative immunotherapy combinations, presenting promising new avenues for the treatment of this aggressive malignancy.

CEP55, a recently identified protein associated with centrosome and midbody, plays a crucial role in cell mitosis. Extensive research has unveiled additional functions of CEP55, including its regulatory roles in the PI3K/AKT pathway and its influence on midbody fate [11, 12]. In cancer biology, CEP55 has gained significant attention in recent years [13]. It has been found to be overexpressed in various malignancies, such as pulmonary cancer, glioblastoma multiforme, head and neck squamous cell carcinoma, and ovarian epithelial tumors, and serves as a prognostic marker for certain cancer patients [14]. Elevated expression of CEP55 has been implicated in the tumorigenesis of diverse cancers, including endometrial cancer, bladder cancer, and clear cell renal cell carcinoma [15–17]. In BC, upregulation of CEP55 has been associated with the malignant growth of cancer cells, exhibiting oncogenic properties [18, 19]. Despite these findings, the specific function of CEP55 in the ferroptosis of BC cells remains largely unexplored.

Our current study has demonstrated the oncogenic role of CEP55 in BC. Moreover, we have identified CEP55 as a critical regulator of ferroptosis in MDA-MB-231 and MCF-7 BC cells. Given that RNA binding proteins (RBPs) play pivotal roles in BC by affecting RNA stability and modulating gene expression [20], we aimed to investigate a BC-associated RBP that drives the upregulation of CEP55 in this malignancy.

## Materials and methods

### Bioinformatic analysis

We analyzed CEP55 expression in human BC by interrogating the GEO GSE227679 dataset (https://www.ncbi.nlm.nih.gov/search/all/?term=GSE227679%20) and the UALCAN-TCGA database (https://ualcan.path.uab.edu/). We also observed ILF3 transcript expression in BC tumor samples using the UALCAN-TCGA database. The Gene Set Cancer Analysis (GSCA) online web (https://guolab.wchscu.cn/GSCA/#/) was used to observe the expression pattern of CEP55 in different subtypes of BC. The KM Plotter (https://www.kmplot.com/analysis/) and GEPIA (http://gepia2.cancer-pku.cn/#index) databases were used to predict the association between CEP55 or ILF3 expression and overall survival or disease-free survival. The LinkedOmics database (https://www.linkedomics.org/#/) was employed to analyze the correlation between CEP55 expression and ferroptosis-related proteins. The open-access web RBPsuite (http://www.csbio.sjtu.edu.cn/bioinf/RBPsuite/reference_new.html) was utilized to predict the RBPs that potentially bind to CEP55 mRNA.

### Human tissue specimens

We obtained informed consent from all patients before the collection of surgically resected primary BC tumors (*n* = 35) and their corresponding non-cancerous breast tissues (*n* = 35) from GaoZhou People’Hospital. None of these BC patients had undergone any prior treatment. Their clinicopathological parameters were shown in Supplementary Table 1. The fresh samples were stored at -80 °C until use for RNA and protein extraction. All human-related procedures adhered strictly to the ethical guidelines approved by the Institutional Committee at GaoZhou People’Hospital [Approval Number: 20221023Q].

### Cell lines

In this study, we used BC cell lines, including SK-BR-3 (#IM-H301, Immocell, Xiamen, China), MDA-MB-231 (#SNL-073, Sunncell, Wuhan, China), and MCF-7 (#SNL-060, Sunncell). These cells were cultured in McCoy’s 5 A (for SK-BR-3, MedChemExpress, Shanghai, China), L15 (for MDA-MB-231, MedChemExpress), and EMEM (for MCF-7, Sunncell). Each medium was enriched with 10% FBS (Biochrom KG, Berlin, Germany) and 1% Penicillin-Streptomycin (MedChemExpress). For MCF-7 cells, the medium was further enriched with Insulin (Biochrom KG) at 10 µg/mL. As a control, we used human non-tumor mammary epithelial MCF-10 A cells (#IM-H315, Immocell), which were cultivated in Immocell-developed standard growth medium (Immocell). All cell lines were maintained in a humidified incubator containing 5% CO_2_ at 37 °C.

### shRNAs, plasmids, and lentivirus particles

We used the following plasmids (Miaoling, Wuhan, China) in this study: pLV3-U6-CEP55(human)-shRNA-CopGFP-Puro (shCEP55, target sequence: 5’-ctgaacttgaaagcaaaaccaat-3’), pLV3-U6-ILF3(human)-shRNA-CopGFP-Puro (shILF3, target sequence: 5’-gccatgtgatggcaaagcattct-3’), shRNA control vector (shNC), and pLV3-CMV-CEP55(human)-CopGFP (oe-CEP55, primers for target sequence: 5’-ATGTCTTCCAGAAGTACCAAA-3’-forward and 5’-CTACTTTGAACAGTATTCCAC-3’-reverse). Lentivirus particles (10^8^ TU/mL) carrying shILF3 or shNC were designed and produced by HanBio (Shanghai, China).

### Transfection and transduction

Following the supplier’s recommendations (Thermo Fisher Scientific, Milan, Italy), we utilized Lipofectamine 3000 as the transfection reagent to introduce shNC, shCEP55, shILF3, or shILF3 + oe-CEP55 into MDA-MB-231 and MCF-7 cells. The cells were subjected to transient transfection at 37 °C for 6 h, and subsequently cultured for another 24 to 72 h after the media were changed. To establish stable ILF3-depleted MDA-MB-231 cells, we transduced these cells with shILF3 or shNC lentivirus particles and selected for virus-positive cells using puromycin, as detailed in the described protocol [21].

### Subcutaneous xenograft studies and immunohistochemistry

Under the protocols approved by the Animal Care Committee of GaoZhou People’Hospital[Approval Number: 20220825D], we utilized 6-week-old male BALB/c nude mice from Vital River Laboratory (Beijing, China) to evaluate the cytotoxic effects of the ferroptosis inducer erastin and ILF3 depletion in MDA-MB-231 subcutaneous xenografts. To establish subcutaneous xenografts, nude mice were injected subcutaneously with untransfected, shNC-, or shILF3-infected MDA-MB-231 cells (5 × 10^6^ cell/mouse) into their right back. Each group contained 6 mice. Erastin administration (Selleck, Shanghai, China, 10 mg/kg) by intraperitoneal injection began after 7 days and was performed on days 7, 14, and 21. We monitored tumor growth (volume = width^2^ × length × 0.5) every one week. After 28 days, the subcutaneous xenografts were harvested, photographed, and weighed. Subsequently, RNA was extracted from the tumor tissues, and the samples were fixed in formalin for further analysis. As previously described [21], we conducted immunohistochemistry on 4-µm-thick formalin-fixed, paraffin-embedded tissue slices, using rabbit polyclonal to CEP55 (#23891-1-AP, Proteintech, Wuhan, China, 1 to 100), rabbit polyclonal to Ki67 (#27309-1-AP, Proteintech, 1 to 5,000), and mouse monoclonal to 4-HNE (#ab48506, Abcam, Cambridge, UK, 1 to 25). Signals were developed with a DAB Color Development Kit (Solarbio, Beijing, China) before hematoxylin counterstaining.

### Extraction of total protein and RNA

We isolated total protein and RNA from tissue specimens or cultivated cell lines using the RNA/Protein Co-extraction Kit, following the manufacturer’s instructions (Beyotime, Shanghai, China). Protein quantification was carried out by BCA method (Thermo Fisher Scientific). RNA concentration was quantified by spectrophotometically (BMG Labtech, Ortenberg, Germany).

### Immunoblotting

Protein (20 µg/line) was separated on 10% gels, and the resulting gels were transferred to nitrocellulose membranes (Millipore, Molsheim, France). Primary antibodies diluted in TBST were used (overnight, 4 °C): rabbit polyclonal to CEP55 (#23891-1-AP, Proteintech, 1 to 2,000), rabbit monoclonal to SLC7A11 (#12691, Cell Signaling Technology, 1 to 1,000), rabbit monoclonal to ILF3 (#ab92355, Abcam, Cambridge, UK, 1 to 20,000), rabbit monoclonal to GPX4 (#ab125066, Abcam, 1 to 5,000), and rabbit polyclonal to β-actin (#ab8227, Abcam, 1 to 4,000). The anti-rabbit HRP secondary antibody (#ab6721, Abcam, 1 to 10,000) was used at room temperature for 1 h. Blot analysis was conducted using Clarity ECL solution (Bio-Rad, Marnes-la-Coquette, France) and Fluorochem M imaging system (Protein Simple, San Jose, CA, USA).

### Quantitative PCR

Total RNA (1 µg) was subjected to DNase treatment and used as a template for cDNA synthesis with the IScript cDNA Synthesis Kit (Bio-Rad). For quantitative PCR reactions, SYBR Green Mix (TaKaRa, Dalian, China) and specific primers (Supplementary Table 2) were used. These reactions were performed on a Chromo4 System (Bio-Rad). Gene expression levels were normalized to the levels of β-actin transcript, and fold changes were determined using the 2^−ΔΔCt^ formula.

### Colony formation assay

After 24 h of transfection, we plated MDA-MB-231 and MCF-7 cells into Corning 6-well culture plates (Corning Costar, Lindfield, NSW, Australia). The cells were then cultured at 37 °C for 12–14 days to allow colony formation. Following crystal violet staining, we counted the number of colonies with more than 50 cells.

### Cell apoptosis assay by flow cytometry

To assess the impact on cell apoptosis, we stained MDA-MB-231 and MCF-7 cells after 72 h transfection with propidium iodide and Annexin V-FITC, followed the protocols provided by the supplier (KeyGen Biotech, Nanjing, China), using an Apoptosis Assay Kit. Within an hour, we measured the apoptotic rate using a cytomics FC500 (Beckman Coulter, Fullerton, CA, USA).

### Cell invasion and migration assays

After 24 h of transfection, we plated serum-starved MDA-MB-231 and MCF-7 cells into 24-Transwell inserts (Corning Costar) pre-coated with Matrigel for evaluating cell invasion or uncoated inserts for motility assessment. The seeding densities were 1 × 10^5^ cells/well for the invasion assay or 5 × 10^4^ cells/well for the migration assay. Subsequently, the inserts were placed into corresponding 24-well plates containing 650 µL of 10% FBS complete medium. Following a 24-h incubation, cells were stained with crystal violet. Images were captured using an Eclipse TS100 microscope (Nikon, Tokyo, Japan). To quantify the invasive or migratory cells, we counted them in more than five randomly selected fields.

### Measurement of MDA, GSH, and Fe2+ contents

We utilized commercial assay kits: the MDA Assay Kit (Beyotime), GSH Assay Kit (Coibo Bio, Shanghai, China), and Ferrous Iron Colorimetric Assay Kit (Elabscience, Wuhan, China), in accordance with the vendors’ suggestions, to measure the concentrations of MDA, GSH, and Fe^2+^ in MDA-MB-231 and MCF-7 cells transfected as indicated.

### RNA immunoprecipitation (RIP) experiment

To evaluate the relationship between ILF3 and CEP55 mRNA, we utilized the BeyoRIP™ RIP Assay Kit as recommended by the supplier (Beyotime). For this experiment, we used rabbit polyclonal to ILF3 (#19887-1-AP) and Isotype IgG (#30000-0-AP) from Proteintech. Untransfected, shNC-transfected, or shILF3-introduced MDA-MB-231 and MCF-7 cells were lysed to prepare total extractions. Protein A/G beads were pre-incubated with the relevant antibody. The cell lysates were then incubated with bead-antibody complex for 4 h at 4 °C. The precipitated complexes were collected, and the associated RNA was extracted. The abundance of enriched CEP55 mRNA was subsequently determined.

### Analysis of CEP55 mRNA stability

After 48 h of transfection, MDA-MB-231 and MCF-7 cells with shNC or shILF3 were exposed to Actinomycin D (Act D, Beyotime) at a concentration of 50 µg/mL. At 0, 2, 4, 6, and 8 h post-treatment, the treated cells were collected for RNA isolation, and the quantity of the remaining CEP55 mRNA was assessed by quantitative PCR.

### Data analysis

We expressed the data as mean ± SD. For statistical analysis, we utilized a two-tailed *t*-test, Mann-Whitney *U* test, or one-way or two-way ANOVA. Significant differences were determined when *P* values were less than 0.05. We performed the Pearson’s correlation analysis to examine the expression association of CEP55 with ILF3 in BC tumors.

## Results

### Upregulation of CEP55 is observed in human BC, and its depletion impairs the malignant potential of BC cells and promotes their ferroptosis

**Fig. 1:**
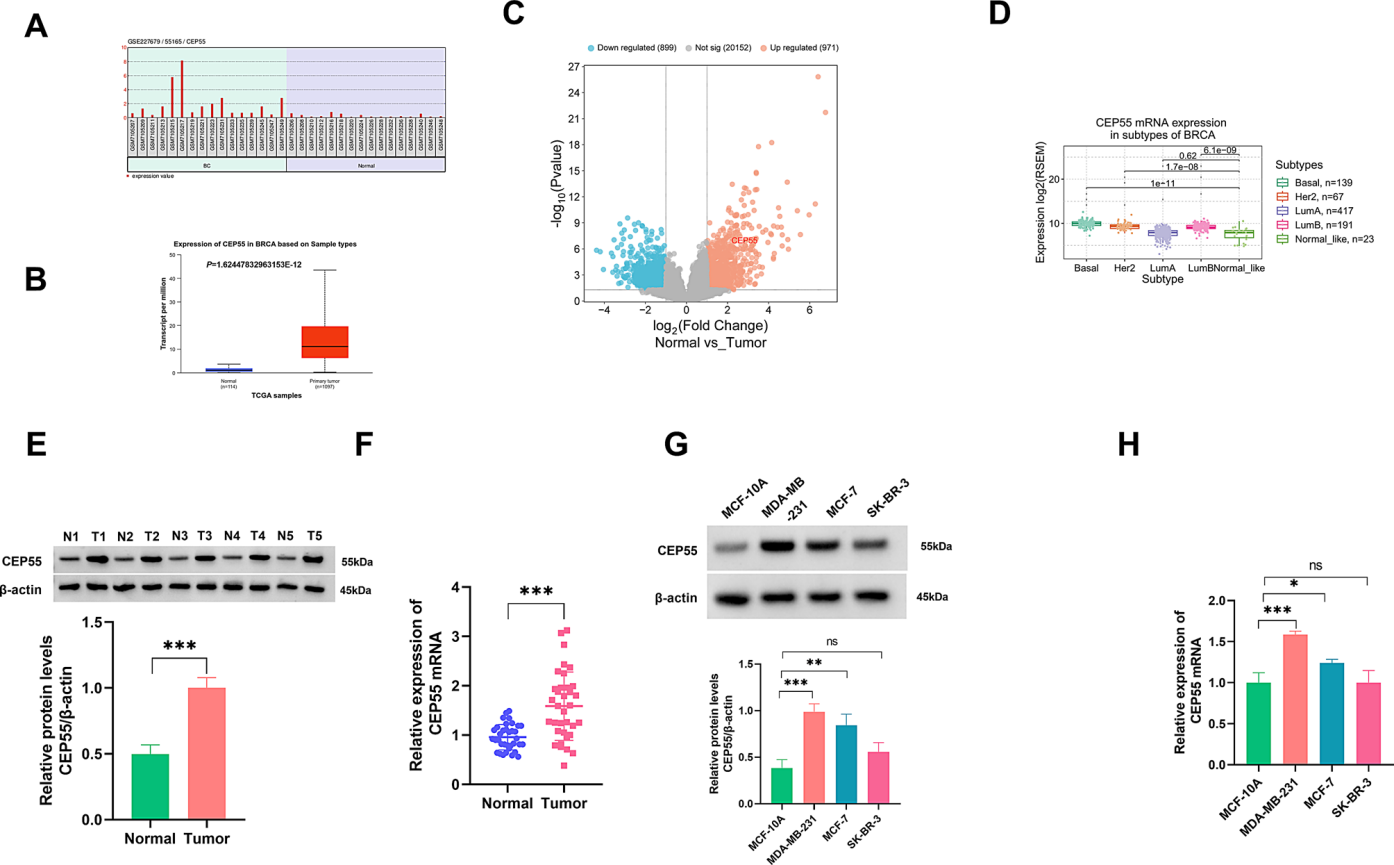
High expression of CEP55 is confirmed in human BC. (**A**-**C**) The GEO GSE227679 dataset and UALCAN-TCGA database showed an upregulation of CEP55 in BC samples compared with their normal counterparts. (**D**) The GSCA online web showed the different expression of CEP55 in different subtypes of BC. (**E**) CEP55 protein expression as analyzed by immunoblot analysis in primary BC tumors (*n* = 3) and matched normal breast samples (*n* = 3). (**F**) CEP55 mRNA expression as examined by quantitative PCR in primary BC tumors (*n* = 35) and matched normal breast samples (*n* = 35). (**G** and **H**) CEP55 protein (immunoblotting) and mRNA (quantitative PCR) expression in MDA-MB-231, SK-BR-3, and MCF-7 BC cells and non-cancerous MCF-10 A cells (*n* = 3). *P < 0.05, **P < 0.01, ***P < 0.001, ns: non-significant

We first analyzed CEP55 expression in human BC using several online computational tools. The GEO GSE227679 dataset and UALCAN-TCGA database showed a strong upregulation of CEP55 mRNA in BC samples compared to their normal counterparts (Fig. 1A and B). Volcano plot of the differentially expressed genes from the GSE227679 dataset revealed the upregulation of CEP55 in human BC (Fig. 1C). The GSCA online web further showed the different expression of CEP55 across various subtypes of BC (Fig. 1D). In addition, the KM Plotter and GEPIA databases showed that in terms of 5-year survival rate, high CEP55 expression predicted a worse overall survival, while CEP55 expression was not significantly associated with disease-free survival in BC patients (Supplementary Fig. 1). We then tested CEP55 expression in clinical specimens from 35 BC patients. Immunoblotting and quantitative PCR showed increased levels of CEP55 protein and mRNA in primary BC tumors compared with normal breast samples (Fig. 1E and F). Furthermore, the protein and mRNA levels of CEP55 were significantly increased in MDA-MB-231 and MCF-7 BC cells, but not in SK-BR-3 BC cells, compared with non-cancerous MCF-10 A cells (Fig. 1G and H).

**Fig. 2:**
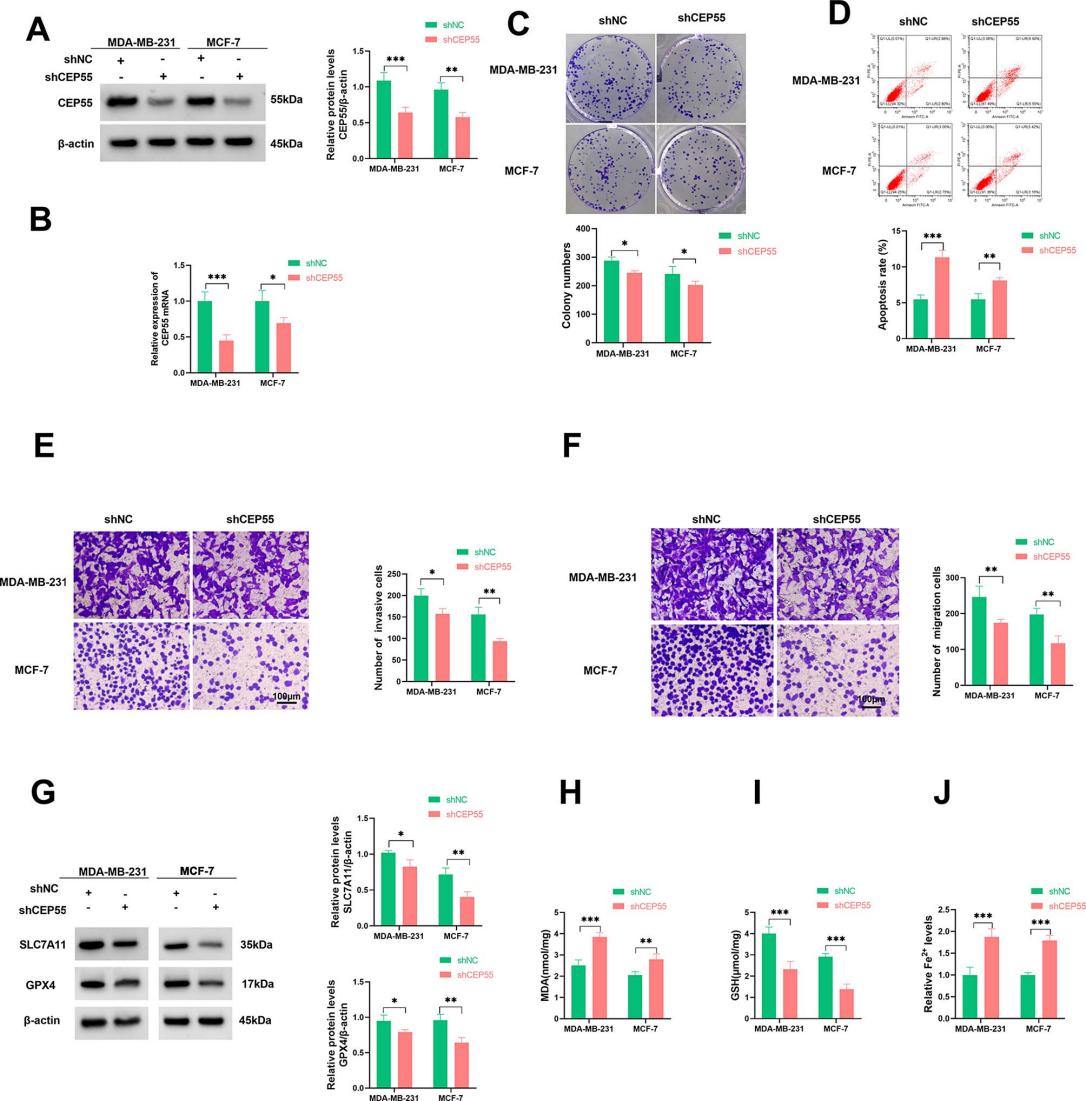
CEP55 depletion impairs cell growth, invasiveness, and motility and enhances cell apoptosis and ferroptosis. (**A** and **B**) Immunoblotting of CEP55 protein expression and quantitative PCR of CEP55 mRNA expression in shNC- or shCEP55-transfected MDA-MB-231 and MCF-7 cells. (**C**) Colony formation ability of CEP55-depleted MDA-MB-231 and MCF-7 cells or control cells. (**D**) Apoptosis rate of CEP55-depleted MDA-MB-231 and MCF-7 cells or control cells as evaluated by flow cytometry. (**E** and **F**) Invasive and migratory capacities of CEP55-depleted MDA-MB-231 and MCF-7 cells or control cells, as examined by transwell assay. Scale bars: 100 μm. (**G**) Immunoblotting of SLC7A11 and GPX4 in CEP55-depleted MDA-MB-231 and MCF-7 cells or control cells. (**H**-**J**) The levels of MDA, GSH, and Fe^2+^ in CEP55-depleted MDA-MB-231 and MCF-7 cells or control cells using the assay kits. *n* = 3 in A-J. *P < 0.05, **P < 0.01, ***P < 0.001

To elucidate the impact of CEP55 upregulation on BC development, we engineered a shRNA-CEP55 (shCEP55) to reduce CEP55 expression in MDA-MB-231 and MCF-7 cells. Immunoblot and quantitative PCR assays confirmed the knockdown efficacy of shCEP55 in the two BC cell lines (Fig. 2A and B). Depletion of CEP55 in MDA-MB-231 and MCF-7 cells caused a reduction in colony formation ability (Fig. 2C), an increase in cell apoptosis rate (Fig. 2D), and impairments in migration and invasion (Fig. 2E and F). Growing evidence unveils the significant role of cancer cell ferroptosis in influencing BC progression [9]. Using the online LinkedOmics database, we found that CEP55 expression was correlated with the levels of ferroptosis-related proteins (SLC7A11, ACSL4, CHAC1, and TFRC) (Supplementary Fig. 2). Thus, we next evaluated the effects of CEP55 depletion on BC cell ferroptosis by measuring related factors. Immunoblot analysis revealed that CEP55-depleted MDA-MB-231 and MCF-7 cells exhibited reduced protein levels of ferroptosis suppressors SLC7A11 and GPX4 compared with shNC controls (Fig. 2G). Furthermore, the levels of MDA and Fe^2+^ were elevated, while GSH content was decreased, in CEP55-depleted MDA-MB-231 and MCF-7 cells (Fig. 2H and J), indicating that CEP55 depletion enhances BC cell ferroptosis. All these results suggest that knocking down CEP55 in CEP55-upregulated BC cells exerts a considerable suppressive effect on their malignant potential.

### ILF3 stabilizes CEP55 mRNA to regulate CEP55 expression in BC cells

**Fig. 3:**
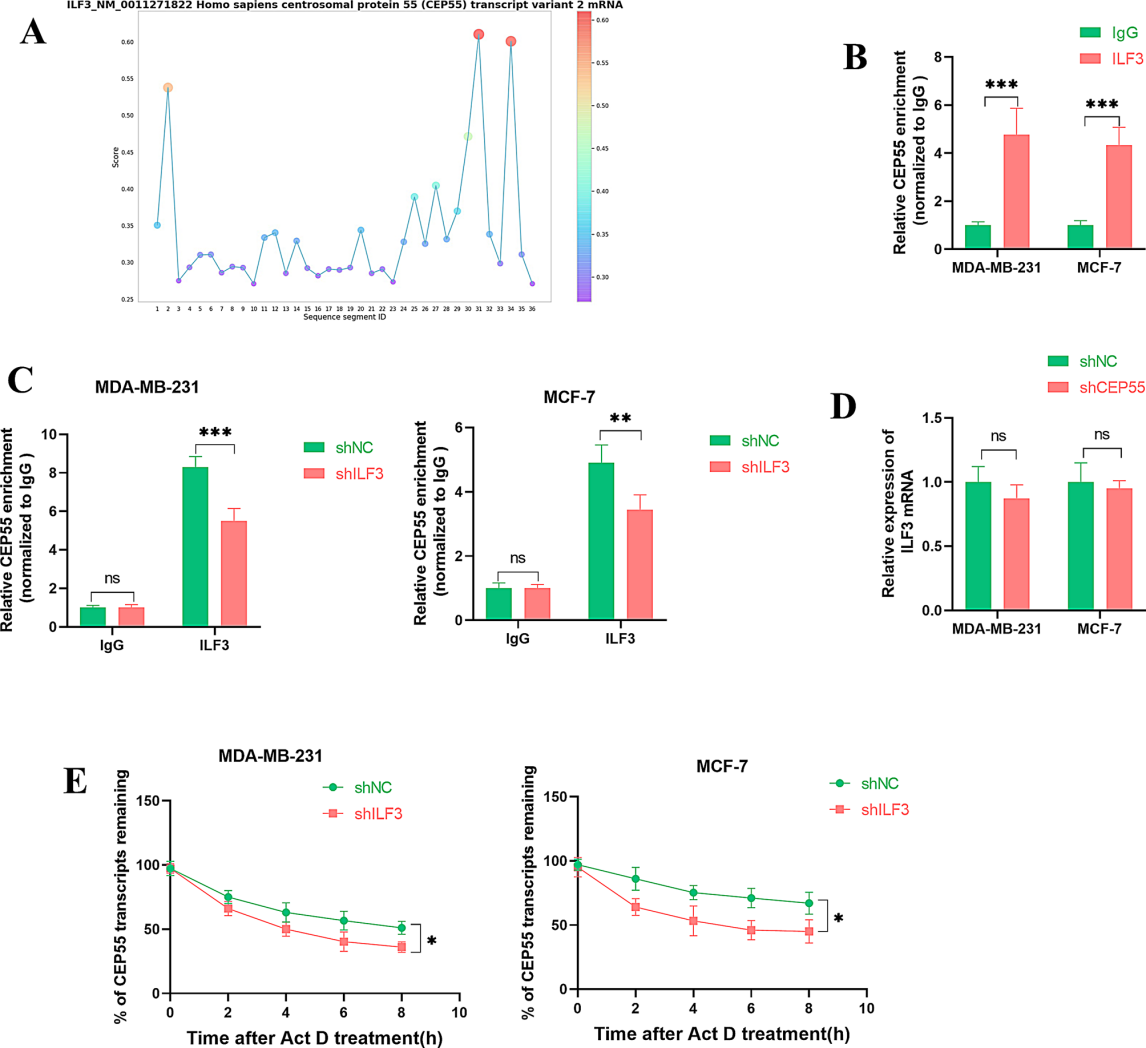
ILF3 affects CEP55 mRNA stability in BC cells. (**A**) The RBPsuite web predicted the potential binding of ILF3 to CEP55 mRNA. (**B** and **C**) RIP experiments using a specific antibody recognizing ILF3 or IgG Isotype in MDA-MB-231 and MCF-7 cells transfected with or without shNC or shILF3. (**D**) Quantitative PCR of ILF3 mRNA in cells transfected with shNC or shILF3 (*n* = 3). (**E**) MDA-MB-231 and MCF-7 cells transfected with shNC or shILF3 were subjected to Act D treatment, followed by detection of the remaining CEP55 mRNA by quantitative PCR. *n* = 3 in B-D. *P < 0.05, **P < 0.01, ***P < 0.001

Next, we sought to identify the regulators driving CEP55 upregulation in BC. RBPs play pivotal roles in controlling RNA metabolism, including RNA stability and degradation, thus modulating gene expression in BC [20]. Thus, we focused on BC-associated RBPs that regulate CEP55 expression in this study. Using the open-access web RBPsuite, we found that RBP ILF3, a strong oncogenic promoter in BC progression [22], had the potential to bind to CEP55 mRNA (Fig. 3A). To investigate this, we carried out RIP experiments using a specific antibody recognizing ILF3 in MDA-MB-231 and MCF-7 cells. By contrast, CEP55 mRNA was strongly enriched in the ILF3-associated precipitates (Fig. 3B), indicating an interaction between ILF3 and CEP55 mRNA. Furthermore, transfection of shRNA-ILF3 (shILF3) into the two BC cell lines led to a striking downregulation in the enrichment levels of CEP55 mRNA (Fig. 3C), supporting the data that ILF3 interacts with CEP55 mRNA in BC cells. Additionally, our data showed that CEP55 did not affect ILF3 mRNA expression in MDA-MB-231 and MCF-7 cells (Fig. 3D). Considering the critical roles of RBPs in mRNA stability, we then examined whether ILF3 could influence the stability of CEP55 mRNA. Using Act D treatment to inhibit transcription, we observed that shILF3 introduction resulted in enhanced degradation of CEP55 mRNA in MDA-MB-231 and MCF-7 cells (Fig. 3E), demonstrating the regulation of ILF3 in CEP55 mRNA stability.

**Fig. 4:**
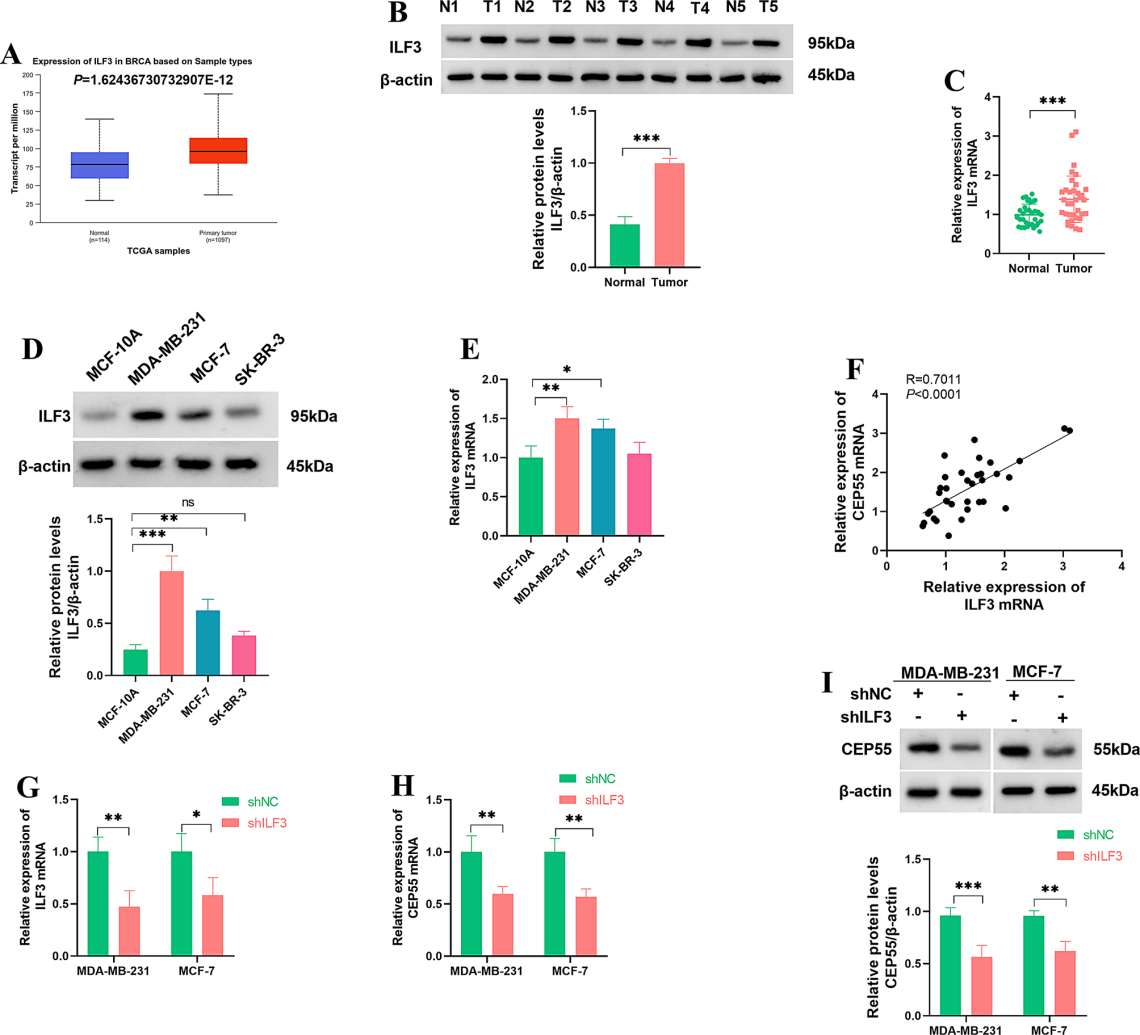
ILF3 regulates CEP55 expression in BC cells. (**A**) The TCGA-BRCA dataset revealed ILF3 increase in BC tumors compared with healthy breast tissues. (**B**) ILF3 protein expression as analyzed by immunoblot analysis in primary BC tumors (*n* = 3) and matched normal breast samples (*n* = 3). (**C**) ILF3 mRNA expression as examined by quantitative PCR in primary BC tumors (*n* = 35) and matched normal breast samples (*n* = 35). (**D** and **E**) ILF3 protein (immunoblotting) and mRNA (quantitative PCR) expression in MDA-MB-231, SK-BR-3, and MCF-7 BC cells and non-cancerous MCF-10 A cells (*n* = 3). (**F**) Pearson’s correlation analysis showed the significant (*P* < 0.0001) and positive (*R* = 0.7011) expression correlation of CEP55 and ILF3 transcripts in primary BC tumors. (**G**) ILF3 mRNA expression as examined by quantitative PCR in MDA-MB-231 and MCF-7 cells transfected with shNC or shILF3 (*n* = 3). (**H** and **I**) Relative CEP55 mRNA and protein levels in MDA-MB-231 and MCF-7 cells transfected with shNC or shILF3 (*n* = 3). *P < 0.05, **P < 0.01, ***P < 0.001, ns: non-significant

The TCGA-BRCA dataset revealed increased expression of ILF3 transcript in BC tumors compared with healthy breast tissues (Fig. 4A). However, the KM Plotter and GEPIA databases revealed that ILF3 expression was not significantly associated with overall survival and disease-free survival in BC patients (Supplementary Fig. 3). Consistent with CEP55 expression patterns, immunoblot and quantitative PCR assays validated elevated levels of ILF3 protein and mRNA in primary BC samples and in MDA-MB-231 and MCF-7 BC cells compared with their normal counterparts (Fig. 4B and E). Intriguingly, a significant (*P* < 0.0001) and positive (*R* = 0.7011) expression correlation of CEP55 and ILF3 transcript levels was observed in primary BC tumors, as evaluated by Pearson’s correlation analysis (Fig. 4F). Transfection of shILF3 into MDA-MB-231 and MCF-7 cells reduced the expression of ILF3 mRNA (Fig. 4G). In contrast, reduced ILF3 expression caused a significant downregulation in CEP55 mRNA and protein levels in the two BC cell lines (Fig. 4H and I). Together, these results demonstrate that ILF3 promotes the stability of CEP55 mRNA, thereby increasing CEP55 expression in BC cells.

### CEP55 restoration partially rescues malignant potential defects of ILF3-depleted BC cells and attenuates their ferroptosis

**Fig. 5:**
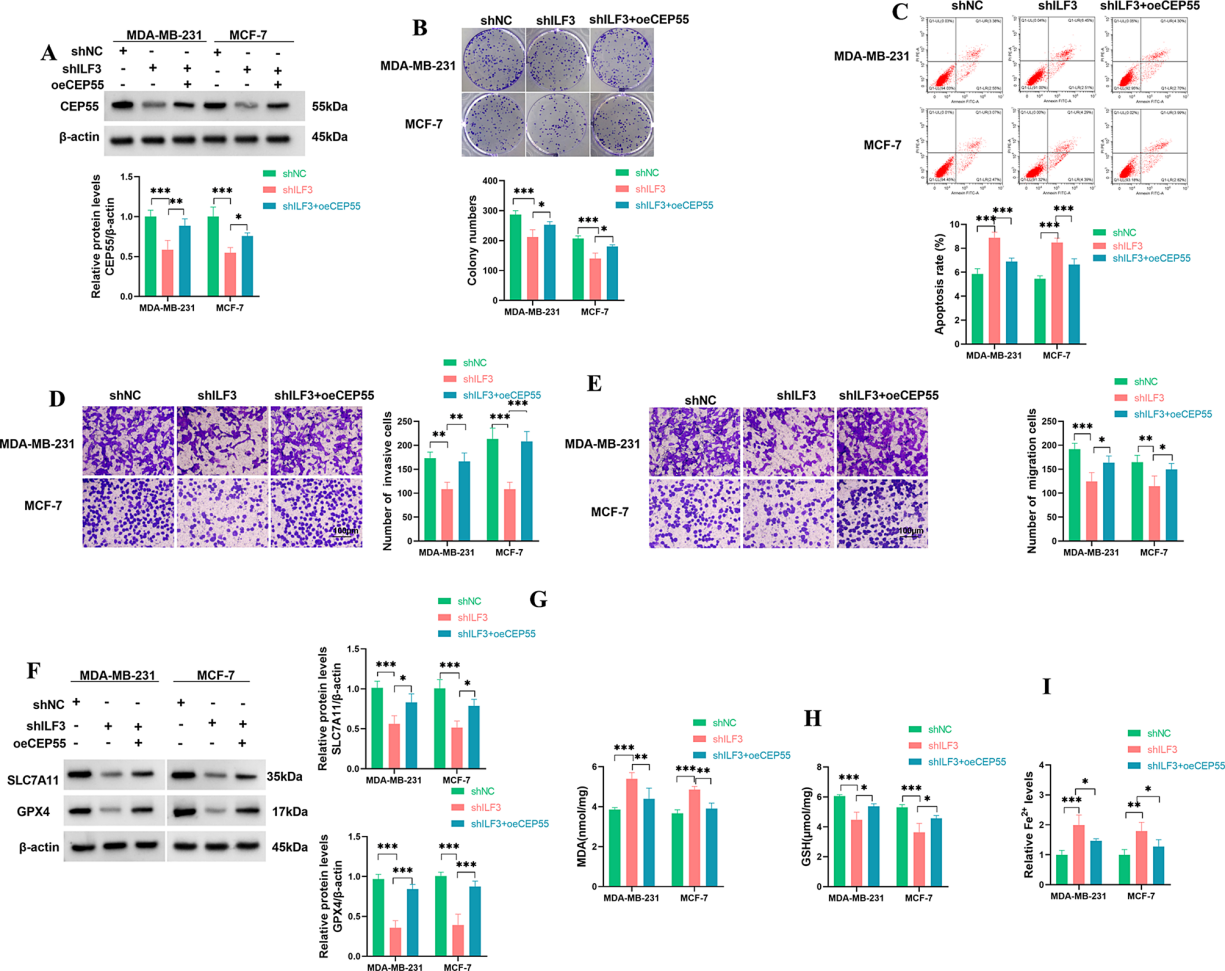
ILF3 affects cell malignant potential and ferroptosis via CEP55. (**A**-**I**) MDA-MB-231 and MCF-7 cells were subjected to transfection with shNC, shILF3, or shILF3 + oeCEP55. (**A**) Immunoblotting of CEP55 protein expression in transfected MDA-MB-231 and MCF-7 cells. (**B**) Colony formation ability of transfected MDA-MB-231 and MCF-7 cells. (**C**) Apoptosis rate of transfected MDA-MB-231 and MCF-7 cells as evaluated by flow cytometry. (**D** and **E**) Invasive and migratory capacities of transfected MDA-MB-231 and MCF-7 cells, as examined by transwell assay. Scale bars: 100 μm. (**F**) Immunoblotting of SLC7A11 and GPX4 in transfected MDA-MB-231 and MCF-7 cells. (**G**-**I**) The levels of MDA, GSH, and Fe^2+^ in transfected MDA-MB-231 and MCF-7 cells using the assay kits. *n* = 3 in **A**-**J**. *P < 0.05, **P < 0.01, ***P < 0.001

Our data showed that in MDA-MB-231 and MCF-7 cells, shILF3-mediated ILF3 decrease caused a downregulation in CEP55 expression (Fig. 5A). To further elucidate the role of the ILF3/CEP55 axis in BC development, we elevated CEP55 expression using a CEP55 ORF construct (oeCEP55) in BC cells with ILF3 depletion (Fig. 5A). By contrast, reduced ILF3 expression diminished cell colony formation ability (Fig. 5B), increased cell apoptosis rate (Fig. 5C), and impaired cell migration and invasion (Fig. 5D and E) in MDA-MB-231 and MCF-7 cells, whereas these effects were partially but significantly reversed by restored CEP55 expression (Fig. 5B and E). Our data also supported the enhancement of ILF3 reduction in cell ferroptosis, as evidenced by decreased levels of SLC7A11, GPX4, and GSH and increased levels of MDA and Fe^2+^ in MDA-MB-231 and MCF-7 cells, which could be partially abolished by CEP55 restoration (Fig. 5F and I). These data indicate that ILF3 affects cell malignant potential and ferroptosis via CEP55.

### ILF3 depletion enhances the anti-tumor growth activity of erastin in MDA-MB-231 subcutaneous xenografts

**Fig. 6:**
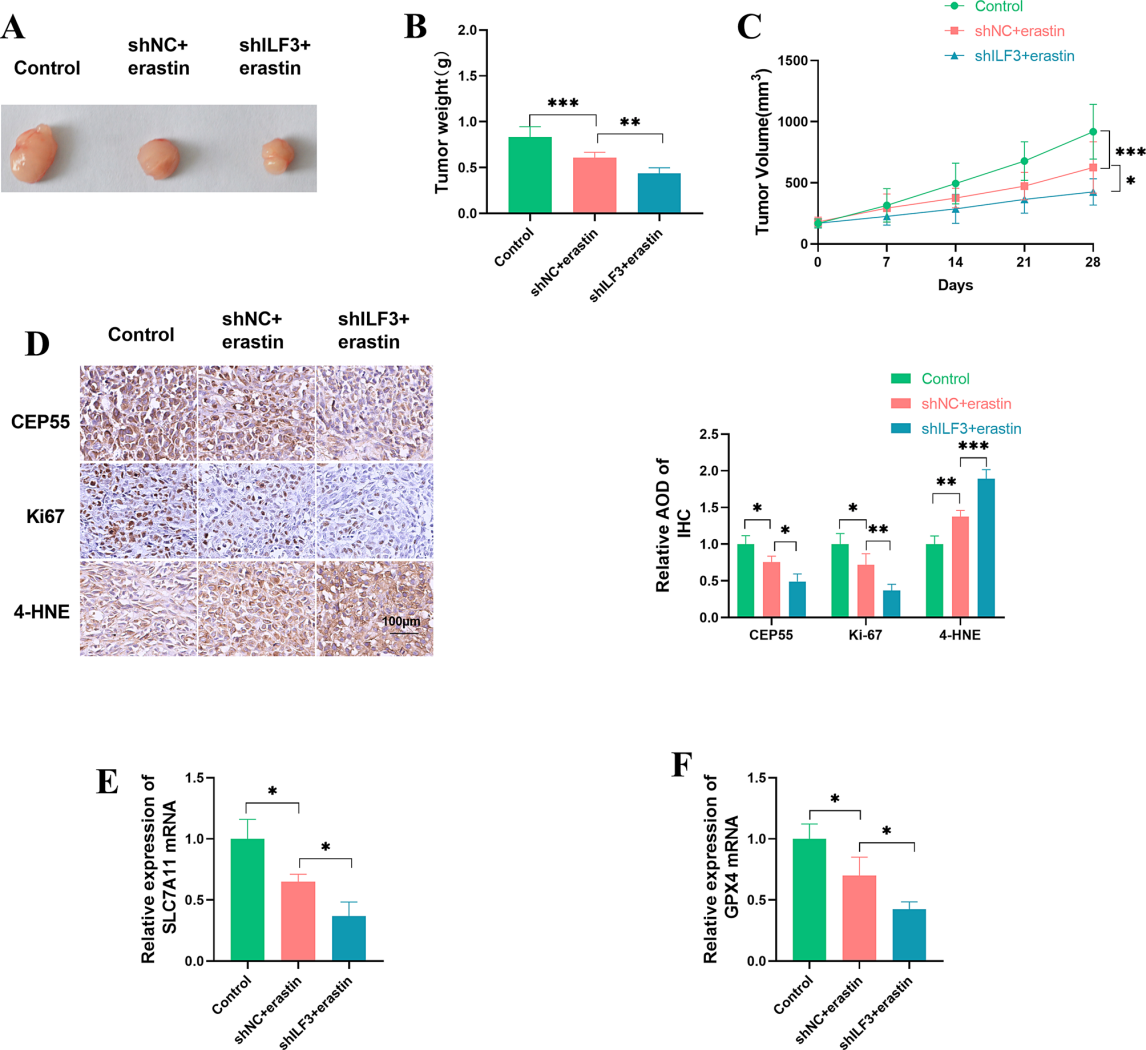
ILF3 depletion enhances the anti-tumor growth activity of erastin in MDA-MB-231 subcutaneous xenografts. (**A**-**F**) Untransfected, shNC- or shILF3-infected MDA-MB-231 cells were implanted into 6-week-old nude mice by subcutaneous injection to produce MDA-MB-231 subcutaneous xenografts. Erastin administration by intraperitoneal injection began after 7 days and was performed on days 7, 14, and 21. After 28 days, subcutaneous xenografts were harvested. *n* = 6 for each group. (**A**-**C**) Representative images (**A**), tumor weight (**B**), and growth curves (**C**) of subcutaneous xenografts. (**D**) Immunohistochemistry of CEP55, Ki67, and 4-HNE in subcutaneous xenografts. (**E** and **F**) Relative mRNA expression of SLC7A11 and GPX4 in MDA-MB-231 subcutaneous xenografts. *P < 0.05, **P < 0.01, ***P < 0.001

We investigated the impact of ILF3 depletion on the growth of MDA-MB-231 subcutaneous xenografts under treatment with the ferroptosis inducer erastin. As shown in Fig. 6A and C, MDA-MB-231 cells successfully formed subcutaneous xenografts in 6-week-old nude mice, whereas erastin administration via intraperitoneal injection reduced tumor growth. Notably, ILF3 depletion caused a significant inhibition in tumor growth under erastin treatment (Fig. 6A and C). Moreover, erastin administration reduced the expression of CEP55, the ratio of Ki67-positive cells, and the mRNA levels of SLC7A11 and GPX4, as well as increased the expression of the ferroptosis inducer 4-HNE in MDA-MB-231 subcutaneous xenografts, while ILF3 depletion exacerbated these alterations (Fig. 6D and F). These results suggest that ILF3 depletion may diminish the growth of MDA-MB-231 subcutaneous xenografts by promoting ferroptosis.

## Discussion

In recent years, BC continues to pose a significant challenge in therapeutic management, despite notable advancements [3]. The emergence of ferroptosis as a promising therapeutic strategy in cancer treatment has garnered considerable attention [6]. In our present study, we have demonstrated that CEP55 plays an oncogenic role in BC by modulating BC cell ferroptosis, thereby contributing to a deeper understanding of its biological significance in BC. Furthermore, we have unveiled a novel regulatory mechanism underlying CEP55 upregulation. Specifically, the RBP ILF3, a strong oncogenic promoter in BC progression [22], drives CEP55 upregulation in BC by enhancing CEP55 mRNA stability.

CEP55 has been increasingly recognized as a potent pro-tumorigenic factor in BC, with its overexpression often observed in BC patient samples. CEP55 deletion can promote the sensitivity of BC cells to anti-mitotic agents [19]. Depletion of CEP55 leads to repressed growth of BC cells by promoting cell cycle arrest and apoptosis [23]. Moreover, reduced CEP55 expression can impair the motility and invasiveness of BC cells [18, 24]. Increased CEP55 expression can enhance the growth and migration of BC cells [13]. Aberrant expression of CEP55 has relevance to the tumor microenvironment and immune regulation in BC [13]. Our study adds to this body of knowledge by confirming the high expression of CEP55 in approximately 80% of BC patients and in MDA-MB-231 and MCF-7 BC cell lines, consistent with previous work [13, 23]. Notably, we have found that depletion of CEP55 significantly impairs the malignant potential of the two BC cell lines, indicating its crucial role in maintaining the tumorigenic phenotype. Accumulating evidence highlights the crucial role that cancer cell ferroptosis plays in modulating the progression of BC [9]. SLC7A11 and GPX4 are key components of the cellular defense mechanism against ferroptosis. SLC7A11 is responsible for the synthesis of the intracellular antioxidant GSH, which works in conjunction with GPX4 to neutralize ROS and prevent lipid peroxidation [6]. MDA is a byproduct of lipid peroxidation and is commonly used as a marker of ferroptosis. Fe^2+^ is a catalyst for the Fenton reaction, which generates ROS and promotes lipid peroxidation, thus driving ferroptosis [25]. By detecting these markers, our results demonstrate, for the first time, that CEP55 depletion promotes ferroptosis of MDA-MB-231 and MCF-7 cells, suggesting a potential mechanism through which CEP55 contributes to BC progression. Given the differential expression of CEP55 across various BC subtypes, our findings highlight CEP55 as a promising personalized therapeutic target in combating BC. Tailoring therapies to inhibit CEP55 in CEP55-overexpressing tumors could represent a novel strategy to enhance treatment efficacy in BC patients. Moreover, we have discovered that CEP55 is significantly upregulated in MDA-MB-231 and MCF-7 cells, but not in SK-BR-3 cells, compared with non-cancerous MCF-10 A cells. We hypothesize that the differential expression of CEP55 in BC cell lines, with the highest levels in the TNBC cell line MDA-MB-231, could be attributed to tumor heterogeneity, the aggressive nature of TNBC, distinct molecular signatures, epigenetic alterations, and unique microenvironmental interactions.

RBPs have emerged as critical regulators in cancer biology, influencing various aspects of tumorigenesis and progression by influencing mRNA stability [26, 27]. Among these, ILF3, a member of the interleukin enhancer-binding factor family, has garnered attention for its pivotal role in human cancers. ILF3 has been found to be upregulated in various cancers, including colorectal cancer, lung cancer and hepatocellular carcinoma [28–30]. Accumulating evidence has highlighted the potential oncogenic activity of ILF3 in multiple malignancies, such as lung cancer, multiple myeloma, and bladder cancer [30–32]. Furthermore, ILF3 has been reported to exert pro-tumorigenic activity in BC, and its depletion can impair BC cell malignant phenotypes, including proliferation, migration, and invasion [22, 33]. Here, we demonstrate that ILF3 stabilizes CEP55 mRNA, thereby regulating CEP55 expression in BC cells. Consistent with the effects observed upon CEP55 depletion and previous findings [22, 33], we also uncover that depletion of ILF3 impairs the in vitro malignant potential of MDA-MB-231 and MCF-7 BC cells and induces their ferroptosis. It is worth noting that restoration of CEP55 expression can partially rescue the malignant potential defects and attenuate ferroptosis in ILF3-depleted BC cells. Thus, our findings reveal that ILF3 contributes to BC development through the upregulation of CEP55. Hu and colleagues have previously unveiled the promoting impact of ILF3 on BC progression by modulating sustained uPA expression [33]. While our study identifies ILF3 as a regulator of CEP55 mRNA stability, further research is needed to elucidate the specific binding sites and mechanisms underlying their interaction. Additionally, it is important to note that our current findings are limited by the smaller subset (only *n* = 5) of western blot data in the human clinical samples (*n* = 35). Further research with a larger sample set for CEP55 and IL3 protein expression will be warranted to determine their abnormal expression in BC patients and whether increased protein levels of CEP55 and IL3 correlate with worse patient survival.

Our findings further indicate that ILF3 depletion enhances the anti-tumor growth activity of erastin in MDA-MB-231 subcutaneous xenografts. Based on the impact on the expression of ferroptosis inhibitors 4-HNE, SLC7A11, and GPX4, ILF3 depletion may diminish the growth of these xenografts by promoting ferroptosis. However, the precise roles of ILF3 and CEP55 in regulating tumor cell ferroptosis in vivo warrants further investigation. Additionally, our results raise the intriguing possibility that shCEP55 and shILF3 could represent promising anti-BC agents. More studies are required to evaluate the safety and efficacy of these potential therapeutic agents using diverse animal models. While our data demonstrate that ILF3 stabilizes CEP55 mRNA in BC cells, the precise binding sites of ILF3 on CEP55 mRNA have not been fully elucidated in this study, which is a big limitation of our current study. Further experiments using RIP followed by sequencing (RIP-Seq) and luciferase reporter assays to identify the specific regions of CEP55 mRNA that interact with ILF3 will be necessary.

In summary, our findings show that ILF3 depletion impairs the malignant potential of BC cells and promotes their ferroptosis through the downregulation of CEP55. Inhibition of ILF3 or CEP55 could represent a promising therapeutic approach for BC.

## Electronic supplementary material

Below is the link to the electronic supplementary material.

Supplementary Material 1: Supplementary Fig. 1. Association between CEP55 expression and overall survival and disease-free survival curves of BC patients. Supplementary Fig. 2. Correlation between CEP55 expression and ferroptosis-related proteins (SLC7A11, ACSL4, CHAC1, TFRC) predicted using the LinkedOmics database. Supplementary Fig. 3. Association between IL3 expression and overall survival and disease-free survival curves of BC patients. Supplementary Table 1. The clinicopathological parameters of BC patients (*n* = 35). Supplementary Table 2. Sequences of quantitative PCR primers

## Data Availability

The analyzed data sets generated during the present study are available from the corresponding author on reasonable request.

## Declarations

### Animal studies

Animal studies were performed in compliance with the ARRIVE guidelines and the Basel Declaration. All animals received humane care according to the National Institutes of Health (USA) guidelines.
