# Supplementary material for: RNA binding protein ILF3 increases CEP55 mRNA stability to enhance malignant potential of breast cancer cells and suppress ferroptosis
Source: Hereditas. 2025 Jan 27;162:10. doi: 10.1186/s41065-025-00372-0 (PMC11773698; doi:10.1186/s41065-025-00372-0)
Supplement: Supplementary file 1 — Supplementary Material 1: Supplementary Fig. 1. Association between CEP55 expression and overall survival and disease-free survival curves of BC patients. Supplementary Fig. 2. Correlation between CEP55 expression and ferroptosis-related proteins (SLC7A11, ACSL4, CHAC1, TFRC) predicted using the LinkedOmics database. Supplementary Fig. 3. Association between IL3 expression and overall survival and disease-free survival curves of BC patients. Supplementary Table 1. The clinicopathological parameters of BC patients (n = 35). Supplementary Table 2. Sequences of quantitative PCR primers [file 41065_2025_372_MOESM1_ESM.docx]

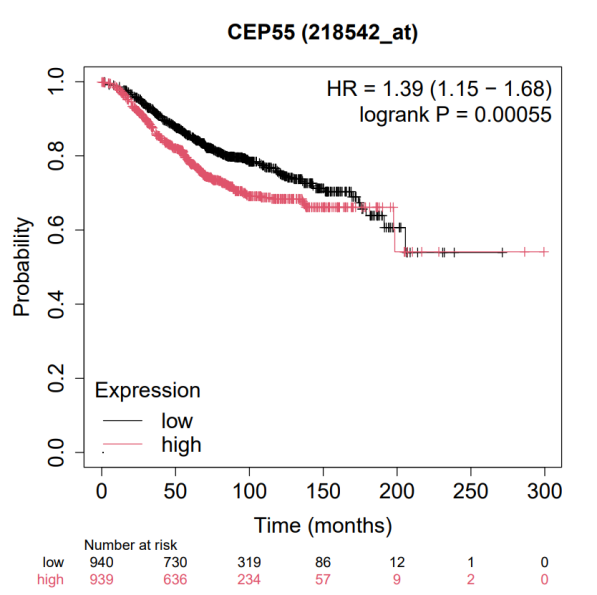

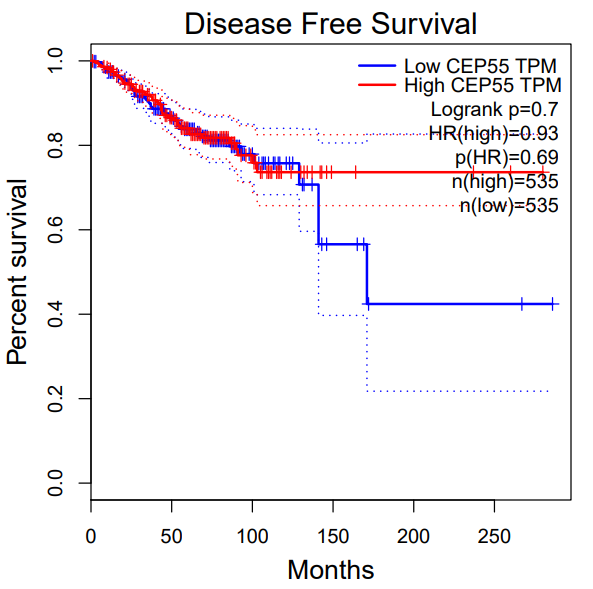


**Supplementary Figure 1.** Association between CEP55 expression and overall survival and disease-free survival curves of BC patients


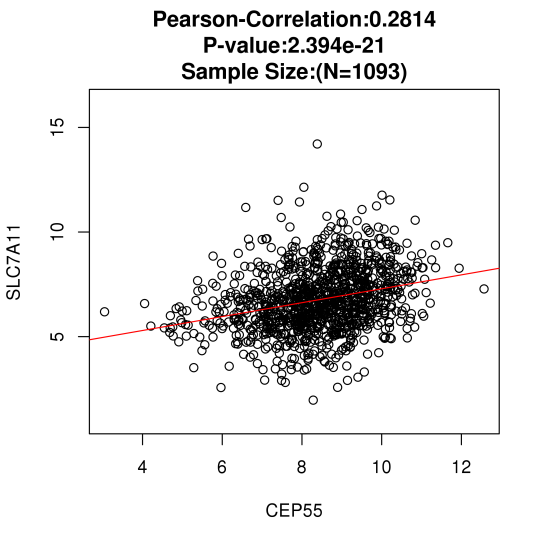

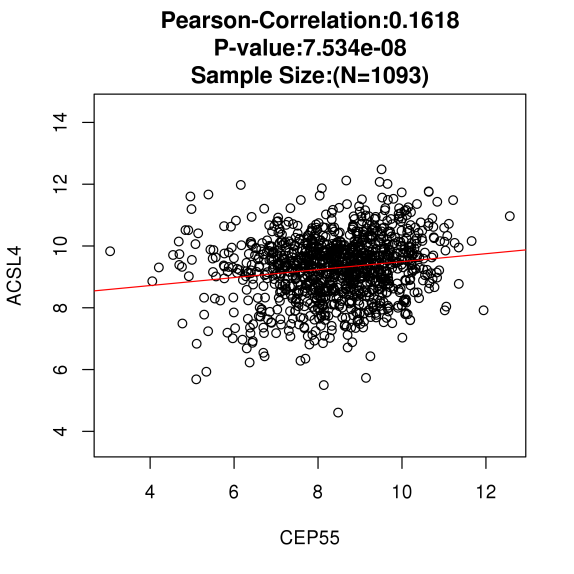


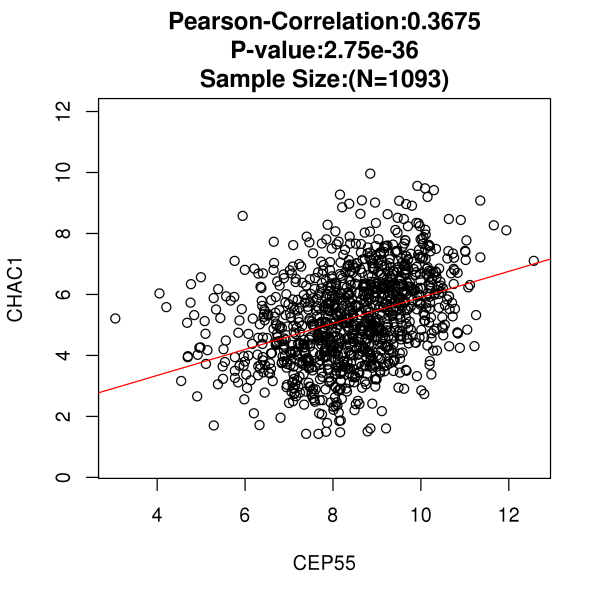

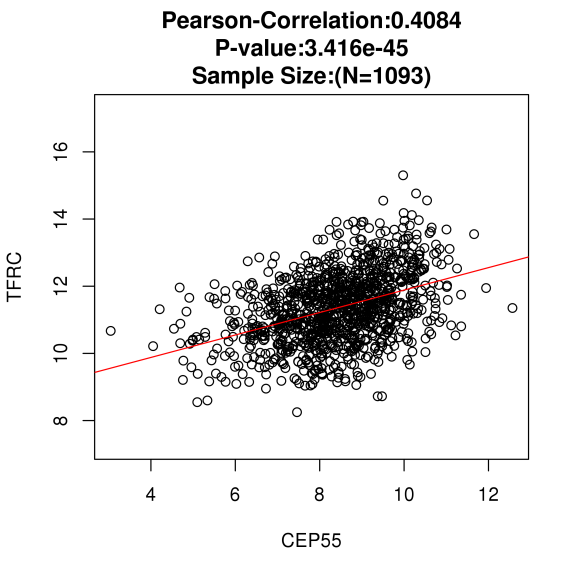


**Supplementary Figure 2.** Correlation between CEP55 expression and ferroptosis-related proteins (SLC7A11, ACSL4, CHAC1, TFRC) predicted using the LinkedOmics database.


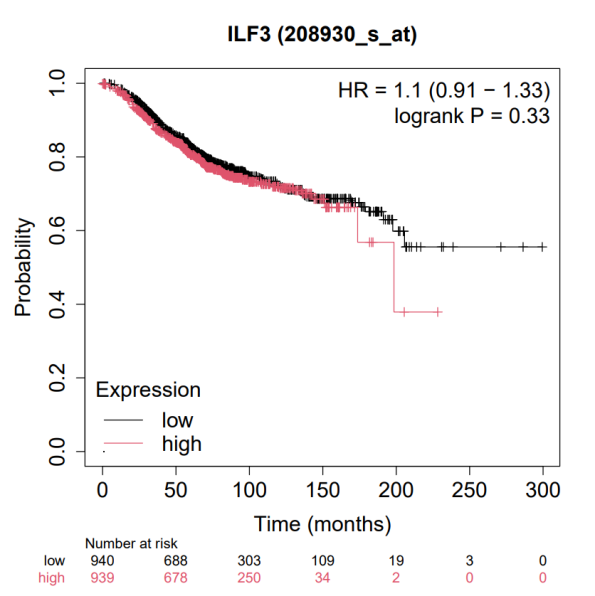

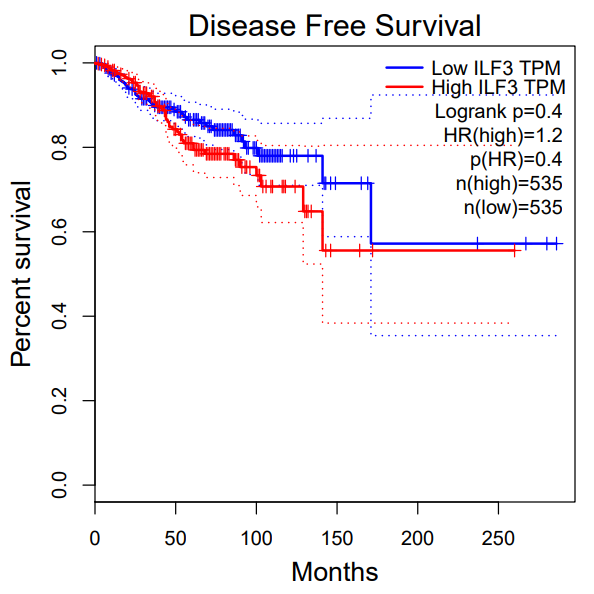


**Supplementary Figure 3.** Association between IL3 expression and overall survival and disease-free survival curves of BC patients

**Supplementary Table 1. The clinicopathological parameters of BC patients (n=35)**

| **Parameters** | **n=35** |
| --- | --- |
| Age (years) |  |
| ≤55 | 24 |
| >55 | 11 |
| Stage |  |
| I | 9 |
| II-III | 26 |
| Lymph node metastasis |  |
| Positive | 12 |
| Negative | 23 |
| Tumor size (cm) |  |
| **<**2 | 7 |
| ≥2 | 28 |
| Histological type |  |
| luminal A | 19 |
| luminal B | 6 |
| HER-2 | 7 |
| Basal-like cancer | 3 |

**Supplementary Table 2. Primers sequences used for quantitative PCR**

| **Name** |  | **Primers for qRT-PCR (5’-3’)** |
| --- | --- | --- |
| CEP55 | Forward | GGGAGGGCAGACCATTTCAG |
|  | Reverse | TACTAGGCTTCGATCCCCAC |
| ILF3 | Forward | CAGATGCCACTGCCGAGC |
|  | Reverse | TTCGACCTCCATGACGAAGC |
| GPX4 | Forward | TCACCAAGTTTGGACACCGT |
|  | Reverse | ATAGTGGGGCAGGTCCTTCT |
| SLC7A11 | Forward | TGGAACGAGGAGGTGGAGAA |
|  | Reverse | TGGTGGACACACAGGCTTT |
| β-actin | Forward | GGATTCCTATGTGGGCGACGA |
|  | Reverse | GCGTACAGGGATAGCACAGC |
